# A Two-color Single-molecule Sequencing Platform and Its Clinical Applications

**DOI:** 10.1093/gpbjnl/qzae006

**Published:** 2024-01-11

**Authors:** Fang Chen, Bin Liu, Meirong Chen, Zefei Jiang, Zhiliang Zhou, Ping Wu, Meng Zhang, Huan Jin, Linsen Li, Liuyan Lu, Huan Shang, Lei Liu, Weiyue Chen, Jianfeng Xu, Ruitao Sun, Guangming Wang, Jiao Zheng, Jifang Qi, Bo Yang, Lidong Zeng, Yan Li, Hui Lv, Nannan Zhao, Wen Wang, Jinsen Cai, Yongfeng Liu, Weiwei Luo, Juan Zhang, Yanhua Zhang, Jicai Fan, Haitao Dan, Xuesen He, Wei Huang, Lei Sun, Qin Yan

**Affiliations:** GeneMind Biosciences Co., Ltd., Shenzhen 518000, China; GeneMind Biosciences Co., Ltd., Shenzhen 518000, China; GeneMind Biosciences Co., Ltd., Shenzhen 518000, China; GeneMind Biosciences Co., Ltd., Shenzhen 518000, China; GeneMind Biosciences Co., Ltd., Shenzhen 518000, China; GeneMind Biosciences Co., Ltd., Shenzhen 518000, China; GeneMind Biosciences Co., Ltd., Shenzhen 518000, China; GeneMind Biosciences Co., Ltd., Shenzhen 518000, China; GeneMind Biosciences Co., Ltd., Shenzhen 518000, China; GeneMind Biosciences Co., Ltd., Shenzhen 518000, China; GeneMind Biosciences Co., Ltd., Shenzhen 518000, China; GeneMind Biosciences Co., Ltd., Shenzhen 518000, China; GeneMind Biosciences Co., Ltd., Shenzhen 518000, China; GeneMind Biosciences Co., Ltd., Shenzhen 518000, China; GeneMind Biosciences Co., Ltd., Shenzhen 518000, China; GeneMind Biosciences Co., Ltd., Shenzhen 518000, China; GeneMind Biosciences Co., Ltd., Shenzhen 518000, China; GeneMind Biosciences Co., Ltd., Shenzhen 518000, China; GeneMind Biosciences Co., Ltd., Shenzhen 518000, China; GeneMind Biosciences Co., Ltd., Shenzhen 518000, China; GeneMind Biosciences Co., Ltd., Shenzhen 518000, China; GeneMind Biosciences Co., Ltd., Shenzhen 518000, China; GeneMind Biosciences Co., Ltd., Shenzhen 518000, China; GeneMind Biosciences Co., Ltd., Shenzhen 518000, China; GeneMind Biosciences Co., Ltd., Shenzhen 518000, China; GeneMind Biosciences Co., Ltd., Shenzhen 518000, China; GeneMind Biosciences Co., Ltd., Shenzhen 518000, China; GeneMind Biosciences Co., Ltd., Shenzhen 518000, China; GeneMind Biosciences Co., Ltd., Shenzhen 518000, China; GeneMind Biosciences Co., Ltd., Shenzhen 518000, China; GeneMind Biosciences Co., Ltd., Shenzhen 518000, China; GeneMind Biosciences Co., Ltd., Shenzhen 518000, China; GeneMind Biosciences Co., Ltd., Shenzhen 518000, China; GeneMind Biosciences Co., Ltd., Shenzhen 518000, China; GeneMind Biosciences Co., Ltd., Shenzhen 518000, China

**Keywords:** GenoCare, Single-molecule sequencing, SARS-CoV-2, Microbial quantitation, Variant detection

## Abstract

DNA sequencers have become increasingly important research and diagnostic tools over the past 20 years. In this study, we developed a single-molecule desktop sequencer, GenoCare 1600 (GenoCare), which utilizes amplification-free library preparation and two-color sequencing-by-synthesis chemistry, making it more user-friendly compared with previous single-molecule sequencing platforms for clinical use. Using the GenoCare platform, we sequenced an *Escherichia coli* standard sample and achieved a consensus accuracy exceeding 99.99%. We also evaluated the sequencing performance of this platform in microbial mixtures and coronavirus disease 2019 (COVID-19) samples from throat swabs. Our findings indicate that the GenoCare platform allows for microbial quantitation, sensitive identification of the severe acute respiratory syndrome coronavirus 2 (SARS-CoV-2) virus, and accurate detection of virus mutations, as confirmed by Sanger sequencing, demonstrating its remarkable potential in clinical application.

## Introduction

Beginning in 1990, the National Human Genome Research Institute of the National Institutes of Health spent 14 years assembling the first version of the human genome [1]. After this landmark achievement, different sequencing techniques have been developed and commercialized to reduce the cost and increase the efficiency [2–9]. The resulting high-throughput and low-cost genomic sequencing techniques, especially next-generation sequencing (NGS) based on clonal amplification and sequencing-by-synthesis (SBS), have revolutionized biomedical research and clinical diagnosis [10–13]. Single-molecule sequencing (SMS) technologies have also been developed. The key feature of this method is the ability to conduct sequencing without amplifying target DNA, thereby avoiding the errors and biases introduced by amplification in NGS platforms [14].

Based on different sequencing principles, the three types of SMS techniques are as follows: true SMS (tSMS; Helicos BioSciences), employing virtual reversible terminator chemistry [5,15]; single-molecule real-time (SMRT) sequencing (Pacific Biosciences), which uses a zero-mode waveguide to monitor fluorescence signals emitted by single DNA polymerase extension [9,16]; and nanopore sequencing (Oxford Nanopore Technologies), which measures the current change when DNA is translocated through protein pores [8,17]. Although SMRT and nanopore sequencing can generate long reads (> 10 kb), their applications are limited by low read numbers, high reagent costs, and complicated sample preparation processes. Particularly for clinical applications such as non-invasive paternal test (NIPT) and pathogen detection by metagenomics sequencing, their costs and lengthy protocols make them less attractive compared to NGS sequencing.

Here, we developed an SBS-based SMS platform, which demonstrates high read throughput and accuracy without requiring amplification. In contrast to tSMS, our GenoCare platform is based on two-color chemistry, which improves reaction efficiency, reduces sample-to-data time, and lowers the reagent costs. The 16-channel flow cell could deliver more than 300 million reads per cell, which is one to two orders of magnitude higher than SMRT and nanopore sequencing. The sample preparation and sequencing can be completed in 24 h. Recently, the application of GenoCare in NIPT and tuberculosis studies has been reported [18,19]. For NIPT, SMS improves the detection of common fetal aneuploidies by reducing the GC bias introduced during library preparation and sequencing [19]. For *Mycobacterium tuberculosis* mutant re-sequencing (250× genome coverage), it has been found that mutations in the *gyrA* and *gyrB* genes are the main mechanisms of gatifloxacin resistance [18]. To investigate the performance of the GenoCare platform in pathogen detection, we sequenced the *Escherichia coli* genome, microbial mixtures, and coronavirus disease 2019 (COVID-19) patient samples from throat swabs. The *E. coli* sequencing was finished within 15 h with an average read length of 53 bp and consensus accuracy exceeding 99.99%. Microbial mixture sequencing showed the quantitative response of the sample concentrations. The straightforward workflow and reduced costs demonstrated in this study suggest that the GenoCare platform might be well-suited for clinical applications.

## Results

### Platform and workflow of two-color SMS

#### Two-color SMS chemistry

We developed four “virtual” reversible terminators with 3′-OH unblocked and the inhibition structure on the side chain extended from the base ring (Figure S1A) for SBS with SMS. Every terminator was labeled with a green or red dye, with peak fluorescent emission wavelengths at 552 nm or 664 nm, respectively. For the SBS technique, polymerase inhibition is a critical parameter for terminator nucleotides. We developed a solution reaction method with different oligo templates to test the inhibition performance of terminators. When the oligo templates were hybridized with carboxyfluorescein-labeled primers, the terminators, polymerase, and reaction buffers were added to incorporate the terminators at the end of primers. After a whole reaction, the product was analyzed using capillary electrophoreses at one-base resolution. By controlling reaction conditions, we ensured that only one terminator (adenosine, A) was added to the primer, and the addition of one terminator increased the primer’s mobility from 53 to over 60 due to the side-chain modification structure on the terminator (Figure S1B–D). The reactions were conducted using TTT or TC templates, and the results showed that only one terminator (A) was incorporated (Figure S1C and D). We also introduced terminators A and T to the reaction system with a TA template, and only one terminator was incorporated (Figure S1E). With this solution reaction model, we designed a series of structures, measured their inhibition performance, and selected the most effective structures for each terminator. Another key parameter to consider was the fluorescence intensity of the labels. To optimize the label structure, the properties of dyes were evaluated, including photostability, hydrophobicity, and quantum efficiency [20]. Considering the redox properties of four nucleotide bases [21], *e.g.*, the photoinduced electron transfer effect of G [22] which decreases the fluorescence signal intensities, the cleavable linker in Figure S1A was designed to mitigate the quenching effects of the bases.

Additionally, we improved the incorporation of terminators by the polymerase. Previously, the wild-type Klenow Fragment with Mn^2+^ as a divalent cation was used with virtual terminators (Figure S1A). However, Mn^2+^ could cause a high probability of misincorporation, leading to a high error rate or even complete termination owing to mismatch events. Therefore, we engineered various polymerases and selected those that could catalyze our terminator reaction with Mg^2+^, which exhibited higher efficiency and fidelity.

**Figure 1:**
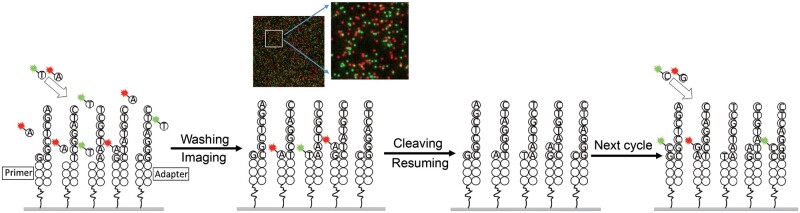
Sequencing cycle scheme

During each sequencing cycle, two terminators with different colors were added and incorporated into primers on the surface. The inhibitor structure guaranteed that there was only a single terminator complementary to the template extension. After washing off unreacted terminators, surface images were captured under the protection of the imaging buffer. The inhibitor structure and dye were cleaved to restart the next cycle (**Figure 1**), in which the other two terminators were incorporated. Different sequencing cycles were selected for different applications to optimize overall reaction time, reagent cost, and read length. To systemically compare the performance of the updated chemistry in sequencing, one library was constructed with a human sample (HCT116) and split into two aliquots for sequencing. Remarkably, after only 72 cycles, the two-color reaction system achieved a read length of ∼ 41 nt, which was 2 nt longer than the read length of the single-color reaction system after 120 cycles, while still yielding the same 10 million (M) mapped reads (Table S1). The updated two-color single-molecule reaction considerably increased the sequencing reaction efficiency. In addition, the reduced reaction cycles and increased hydrophilicity of the new nucleotide resulted in a lower insertion ratio (Figure S2). The error distributions were changed by the new chemistry, and the total error ratio decreased. Compared with NGS systems, which typically require more than 20 reagents for processes including amplification, denaturing, complementary strand removal, and rehybridization of sequencing primers, SMS uses only seven reagents to complete sequencing, substantially reducing the sequencing cost. SMS also mitigates the pre-phasing and phasing issues caused by asynchronous reaction in amplified cluster, which complicate NGS sequencing chemistry and data analysis.

#### Simplified library preparation process

**Figure 2:**
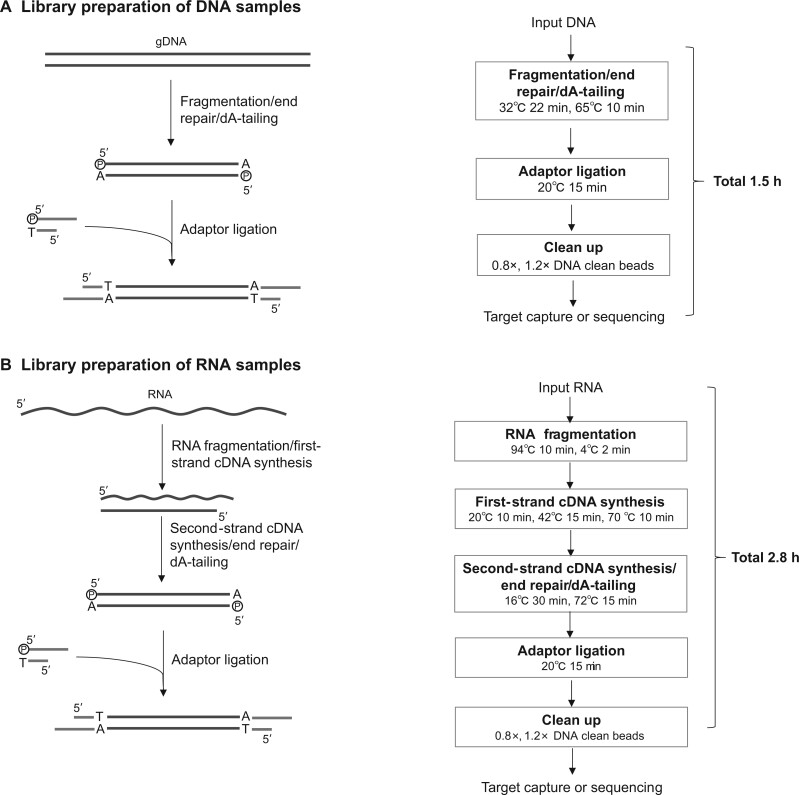
Universal workflow of library preparation for SMS **A**. Library preparation of DNA samples. **B**. Library preparation of RNA samples. dA-tailing means adding a non-template dA to the 3′ end of a blunt-ended DNA fragment. SMS, single-molecule sequencing; gDNA, genomic DNA; cDNA, complementary DNA; dA, deoxyadenosine.

One major advantage of GenoCare over amplification-based NGS technology is its simple library preparation process. **Figure 2** shows the general workflows for the SMS platform’s library preparation, which took only 1.5 h for DNA and 2.8 h for RNA samples, respectively. The GenoCare platform’s library preparation shortened the protocol by allowing single-end ligated adaptor libraries and eliminating the need for indexes. Compared with traditional NGS platforms that could only amplify libraries with double-end ligated adaptors, the GenoCare sequencer requires only one adaptor to hybridize on the flow cell, and sequencing is initiated immediately after the hybridization. This makes it particularly suitable for small quantities of material, like cell-free DNA, requiring as little as 3 ng of DNA to suffice with GenoCare’s library preparation method.

### Performance of SMS in single and mixed microbes

The sequencing of simple genome (phi X174 phage) took 24 h. A total of 334.3 M mappable reads were acquired from 16 lanes (Figure S3), with an average of 20.9 M unique reads and a coefficient of variation of 4.5% per lane.

**Figure 3:**
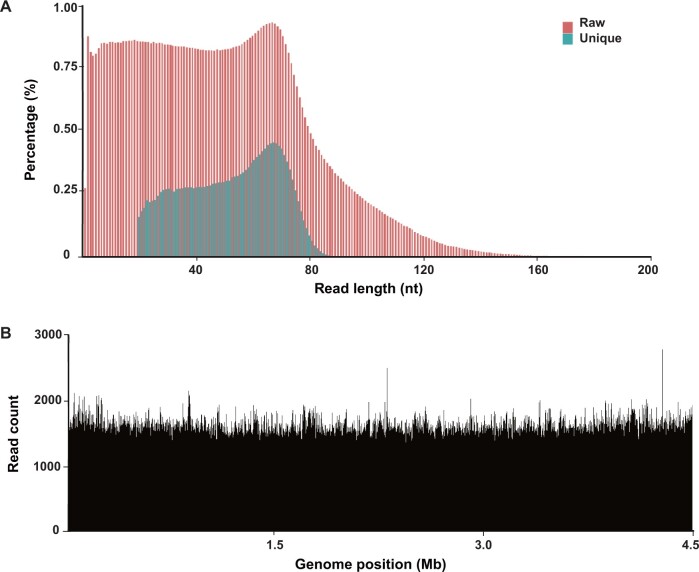
SMS of *E*. *coli* sample **A**. Read length distribution of raw data and unique mapped data. **B**. Coverage plot across the *E*. *coli* genome. *E*. *coli*, *Escherichia coli*.

For the model bacteria (*E. coli*), the entire sequencing process took 15 h. The read length of the highest abundant reads was 70 bp, and the average read length was 53 bp (**Figure 3**A). Each lane yielded 12.2 M unique mapped reads, with a raw mismatch rate of 0.61%, an insertion rate of 1.45%, and a deletion rate of 2.76%. The sequencing depth reached 130× (Figure 3B), with a genome coverage of 99.94% and a consensus accuracy of 99.99%. For the pure *E. coli* concentration gradient experiment, when the concentration was higher than 0.0005%, the ratio obtained by sequencing was close to the theoretical value.

To demonstrate the potential of the SMS platform in clinical metagenomic samples, a mixture of *E. coli*, *Staphylococcus aureus*, phage M13 (circular form), yeast, and human genomic DNA (gDNA) was analyzed (Figure S4; Table S2). The results from the GenoCare platform were consistent with the concentration when the ratio of M13 changed from 1.00E−6 to 1.00E−3 (**Table 1**), which was better than quantitative polymerase chain reaction (qPCR). In contrast, the dynamic range on the HiSeq was from 1.00E−7 to 1.00E−3. Nevertheless, we found that the turnaround cycle on SMS was shorter than that on HiSeq, while the latter also had a higher reagent cost. This model test indicates that GenoCare has the potential to be applied in pathogen detection, though improvements such as pathogen enrichment are necessary to reach a sensitivity comparable to NGS.

**Table 1:** Test read ratio of M13 on GenoCare and HiSeq 4000 sequencers

**Sample**	Theoretical value	qPCR	HiSeq 150 bp	HiSeq 40 bp	GenoCare
PM_A	1.00E–07	2.06E–08	9.58E–08	5.36E–08	6.60E–07
PM_B	1.00E–06	2.64E–07	5.24E–07	5.42E–07	7.70E–07
PM_C	5.00E–06	1.30E–06	2.61E–06	2.66E–06	2.23E–06
PM_D	1.00E–05	3.37E–06	8.96E–06	9.20E–06	4.76E–06
PM_E	5.00E–05	2.65E–05	5.55E–05	5.66E–05	3.31E–05
PM_F	1.00E–04	5.25E–05	1.34E–04	1.37E–04	7.80E–05
PM_G	1.00E–03	4.76E–04	1.41E–03	1.45E–03	8.09E–04

*Note*: Hiseq 150 bp means the 150-bp reads originating from NGS sequencer Hiseq 4000 in SE150 model. HiSeq 40 bp means that the first 40 bp from the Hiseq 150 bp was trimmed. GenoCare means the reads originating from GenoCare 1600.  qPCR, quantitative polymerase chain reaction.

### Accurate detection of SARS-CoV2 mutants

While SMS demonstrated strong performance in single phage, bacterial genome, and mixed microorganisms, we investigated its suitability for tracking severe acute respiratory syndrome coronavirus 2 (SARS-CoV-2). Throat swab samples from three COVID-19 patients and one negative sample were sequenced using the SMS platform. Figure S5 shows the mapped reads across the SARS-CoV-2 genome. Positive samples exhibited a large number of reads that could be uniquely mapped to the SARS-CoV-2 genome from the National Center for Biotechnology Information database compared with the negative sample. Furthermore, with more than 50,000 reads mapped to the virus, we detected that the unique mapped reads covered over 99.9% of the genome (Figure S5). The missed regions belonged to the poly(A) sequences in the virus genome. Mismatch positions detected with a depth of over 90× were similar to those identified by Sanger sequencing (**Table 2**, Tables S3 and S4) and were previously described mutations [23]. We discovered a T-to-C mutation at location 28,144 in the *ORF8* gene, which had a population frequency of 35.98% at that time according to the National Genomics Data Center (https://ngdc.cncb.ac.cn/ncov/variation/statistics). Overall, the findings demonstrate the capability of our technology to detect and monitor pathogenic infections.

**Table 2:** SARS-CoV-2 mutations detected by GenoCare and confirmed by Sanger sequencing

Sample ID	Position (nt)	Mutation	Sequencing depth (×)	Sanger sequencing verification
1022T	1397	G>A	969	A
	11,083	G>T	129	A (reverse)
	28,688	T>C	1118	G (reverse)
	29,742	G>T	294	T
	29,776	A>T	95	T
1028T	8782	C>T	290	A (reverse)
	8937	C>T	167	A (reverse)
	28,144	T>C	231	C
	28,878	G>A	239	A
	29,742	G>A	184	A
1016T	14,342	T>C	39	C
	25,095	C>T	27	T
	28,030	T>C	59	C

*Note*: SARSCoV-2, severe acute respiratory syndrome coronavirus 2.

## Discussion

The GenoCare sequencer has been designed and developed for clinical applications based on the single-molecule SBS technique, which exhibits strong performance in the field of reproductive health, such as NIPT, preimplantation genetic testing, and chromosome analysis of miscarriage tissues, owing to its easy library preparation, low facility demand, and high sensitivity [24,25]. The high cost per run, long hands-on operation time, and cumbersome processes for many NGS-based clinical tests might impede their adoption in hospitals, whereas the SMS platform offers advantages in all these respects. Furthermore, the design of 16 physically distinct lanes and independent loading fluidics reduces sample mixing for each run and allows customizable sample sequencing, accommodating more than 16 test samples through the development of two indexes allowing for a maximum of 32 samples per run.

As described in this study, the GenoCare sequencer has shown promise in microbial sequencing with its rapid sample-to-sequencing process and low reagent costs. In addition, it can eliminate errors and biases caused by the NGS amplification process. Currently, the use of the GenoCare sequencer is limited by its relatively high error rate. Our ongoing efforts focus on enhancing the base throughput and accuracy of the sequencer to further improve its performance. This involves the development of flow cell with higher signal density, optimization of nucleic acid and enzyme chemistry to achieve longer read lengths, and refinement of Q-score, base-calling algorithm, and bioinformatics software to decrease the error rate. We anticipate that this platform will expedite the integration of DNA sequencing into clinical diagnostics in the future.

## Conclusion

We have demonstrated that two-color sequencing chemistry improves the efficiency of SMS. The resulting GenoCare sequencing platform exploits new virtual terminator reagents to simplify the sequencing process and reduce reagent costs compared to NGS. This platform enables rapid and accurate quantification of pathogenic bacteria and RNA viruses. Moreover, it shows excellent variant detection in SARS-CoV-2 identification. SMS may offer unique advantages for clinical institutions that lack the facilities and personnel for traditional amplification-based NGS instruments.

## Materials and methods

### Reversible terminator synthesis

Reversible terminators with the structure shown in Figure S1A were synthesized according to a previously reported method [26].

### Reversible terminator assay

A set of 78-nt oligos, sharing the same base sequence except for bases 18 to 20 (5′ to 3′), were synthesized as template oligos by Sangon Biotech (Shanghai) Co., Ltd. The three different bases were designed for different performance tests. The quenched solution (1 µl), mixed with Liz500 standard (0.1 µl) and Hi-Di formamide (0.89 µl), was loaded into the analyzer (Figure S1B–E).

### Sequencing library preparation

The phi X174 phage genome and *E. coli* DNA (ATCC8739) were used to construct libraries following the procedure outlined in Figure 1A.

### Surface hybridization

The surface chemistry of the SMS platform’s flow cell has been described in previous publications [27,28]. Primers immobilized on the flow cell surface were designed to capture the samples with sequencing adaptors.

### Sequencing reagent kit

The GenoCare sequencing reagent kit was utilized, which contained two sets of nucleotide–polymerase mixtures: one for fluorescence dye-labeled A and T virtual terminators, and the other for G and C virtual terminators labeled with the same pair of dyes.

### Sequencer hardware

The GenoCare platform (GeneMind, Shenzhen, China), a desktop platform for two-color SMS, was employed for sequencing. This instrument uses wide-field total internal reflection fluorescence (TIRF) optics to detect weak signals from single-nucleotide molecules.

### Data processing

Raw sequencing image files were analyzed in real-time by our in-house machine learning algorithm-based software (DirectCall 2C0.5.8). The data processing workflow included background elimination, spot localization, image registration, template building, and base-calling.

### Characterization of SMS

Phi X174 phage and *E. coli* genome were sequenced as standard reference templates to characterize the read yield of our platform.

### Detection of microbes

Pure *E. coli* DNA (ATCC8739) and healthy female individual gDNA were mixed to obtain different ratios of *E. coli*. Fourteen libraries were constructed to evaluate the sequencing accuracy for each sample (Table S5). In addition, a mixture of *E. coli* DNA (ATCC8739), *S. aureus* DNA [CMCC(B)26003], M13mp18 RF I DNA (a circular form of phage M13 DNA), yeast DNA, and human DNA from the healthy female individual at different ratios (Table S2) was split into two aliquots, one for the SMS platform and the other for the NGS sequencer (HiSeq 4000, Illumina, San Diego, CA) in SE150 mode.

### Detection of SARS-CoV-2 mutations

In collaboration with Shenzhen Uni-medica Technology Co., Ltd., China, three positive samples (∼ 4 ng/µl) from COVID-19 patients’ throat swabs and one negative sample were acquired. A complementary DNA (cDNA) library was prepared by reverse transcription, and sequencing adaptors were connected to cDNA fragments via the transposase (GenoCare DirectPrep RNA Library Prep Kit, GeneMind) by the procedure shown in Figure S6. After qualification, the libraries were loaded into the GenoCare SMS sequencer for sequencing with 72 cycles and 480 photograph each field of view (FOV) were captured from each cycle.

Extended experimental procedures are available in File S1.

## Ethical statement

Ethical approval and consent to participate in this study were approved by the GeneMind Biosciences Co., Ltd. Ethics Committee (Approval No. GM20220830A). All methods were performed in accordance with relevant guidelines and regulations.

## Supplementary Material

qzae006_Supplementary_Data

## Data Availability

All SMS raw data have been deposited to the Genome Sequence Archive and Genome Sequence Archive for Human [29] at the National Genomics Data Center, Beijing Institute of Genomics, Chinese Academy of Sciences / China National Center for Bioinformation (GSA: CRA010039; GSA-Human: HRA004015), and are publicly accessible at https://ngdc.cncb.ac.cn/gsa and https://ngdc.cncb.ac.cn/gsa-human, respectively.

## CRediT author statement

**Fang Chen:** Conceptualization, Methodology, Writing – original draft, Writing – review & editing. **Bin Liu:** Conceptualization, Methodology. **Meirong Chen:** Methodology, Investigation. **Zefei Jiang:** Methodology. **Zhiliang Zhou:** Methodology. **Ping Wu:** Methodology, Investigation. **Meng Zhang:** Methodology, Investigation. **Huan Jin:** Software, Formal analysis. **Linsen Li:** Software, Formal analysis. **Liuyan Lu:** Methodology, Investigation. **Huan Shang:** Methodology, Investigation. **Lei Liu:** Methodology, Investigation. **Weiyue Chen:** Software, Methodology, Formal analysis. **Jianfeng Xu:** Methodology, Investigation. **Ruitao Sun:** Methodology, Investigation. **Guangming Wang:** Methodology, Investigation. **Jiao Zheng:** Methodology, Investigation. **Jifang Qi:** Methodology, Investigation. **Bo Yang:** Methodology, Investigation. **Lidong Zeng:** Software, Formal analysis. **Yan Li:** Methodology, Investigation. **Hui Lv:** Methodology, Investigation. **Nannan Zhao:** Methodology, Investigation. **Wen Wang:** Methodology, Investigation. **Jinsen Cai:** Methodology, Investigation. **Yongfeng Liu:** Writing – review & editing. **Weiwei Luo:** Methodology, Investigation. **Juan Zhang:** Data curation, Visualization. **Yanhua Zhang:** Software, Formal analysis. **Jicai Fan:** Methodology, Investigation. **Haitao Dan:** Methodology, Resources. **Xuesen He:** Methodology, Resources. **Wei Huang:** Methodology, Resources. **Lei Sun:** Conceptualization, Writing – original draft, Writing – review & editing. **Qin Yan:** Conceptualization, Supervision. All authors have read and approved the final manuscript.

## ORCID

0000-0003-0028-8967 (Fang Chen)

0000-0002-5051-8006 (Bin Liu)

0000-0002-2600-3256 (Meirong Chen)

0000-0001-6053-9030 (Zefei Jiang)

0000-0002-9003-3177 (Zhiliang Zhou)

0000-0001-8717-3067 (Ping Wu)

0000-0002-7588-7182 (Meng Zhang)

0000-0002-9848-516X (Huan Jin)

0000-0002-9206-3740 (Linsen Li)

0000-0001-8680-1957 (Liuyan Lu)

0000-0002-8703-999X (Huan Shang)

0000-0003-2794-6982 (Lei Liu)

0000-0001-5004-4376 (Weiyue Chen)

0000-0002-5127-2300 (Jianfeng Xu)

0000-0003-4165-6615 (Ruitao Sun)

0000-0001-7833-341X (Guangming Wang)

0000-0001-6996-7453 (Jiao Zheng)

0000-0002-9770-2302 (Jifang Qi)

0000-0002-9033-3479 (Bo Yang)

0000-0001-7189-883X (Lidong Zeng)

0000-0003-4718-5551 (Yan Li)

0000-0002-0252-6706 (Hui Lv)

0000-0003-4733-1053 (Nannan Zhao)

0000-0003-0074-570X (Wen Wang)

0000-0002-8621-096X (Jinsen Cai)

0000-0002-2844-1807 (Yongfeng Liu)

0000-0001-5872-1482 (Weiwei Luo)

0000-0003-3072-9145 (Juan Zhang)

0000-0002-9732-740X (Yanhua Zhang)

0000-0001-8002-5678 (Jicai Fan)

0000-0001-5739-5849 (Haitao Dan)

0000-0001-8809-5638 (Xuesen He)

0000-0001-9849-3883 (Wei Huang)

0000-0001-8837-3183 (Lei Sun)

0000-0001-6748-6234 (Qin Yan)
