# Supplementary material for: A Two-color Single-molecule Sequencing Platform and Its Clinical Applications
Source: Genomics Proteomics Bioinformatics. 2024 Jan 11;22(1):qzae006. doi: 10.1093/gpbjnl/qzae006 (PMC11423845; doi:10.1093/gpbjnl/qzae006)
Supplement: qzae006_Supplementary_Data [file qzae006_supplementary_data.zip › File S1.docx]

**File S1 Extended experimental procedure**

**Reversible terminator synthesis**

Reversible terminators with the structure shown in Figure S1A were synthesized using a previously reported method [27]. Fluorescent dyes with emission in the Cy3 (Atto 532: 2822) and Cy5 (Atto 647N: 2856) channels were either purchased from AAT Bioquest (Pleasanton, California) or prepared in-house. Disulfide linkers were constructed and conjugated to propargylamine-modified nucleotide bases by amine-*N*-hydroxysuccinimide ester coupling and then attached to fluorescent dyes. The final compounds were purified by high-performance liquid chromatography.

**Reversible terminator assay**

To test the polymerase incorporation and inhibition performance of the nucleotide terminators, a set of 78-nt-long oligos sharing the same base sequence, except bases 18 to 20 (5' to 3'), were synthesized as template oligos by Sangon Biotech (Shanghai) Co., Ltd. The three different bases were designed for different performance tests; for example, AAA was used for the inhibition test for terminator T. A 58-nt-long oligo labeled with carboxyfluorescein at the 5' end was added as a primer. The concentration of the template oligo was 0.033 μM, whereas the primer concentration was 3 to 5 times higher according to the reaction system. The reaction buffer contained 20 mM Tris-HCl (pH 8.8), 10 mM KCl, 50 mM NaCl, 0.1% Triton X-100, 10 mM (NH_4_)_2_SO_4_, and 10 mM MgSO_4_. The mixture was heated at 95°C for 5 min, 55°C for 5 min, and then 50 U/ml Klenow Fragment (GeneMind Biosciences, Shenzhen, China) was added and the mixture was kept at 37°C for 5 min. The mixture was immediately placed in a −20°C freezer for more than 30 min to quench the reaction. A Genetic Analyzer (ABI PRISM 3100, Thermo Fisher Scientific, Waltham, Massachusetts) was used to characterize the reaction. The quenched solution (1 µl) mixed with Liz500 standard (0.1 µl) and Hi-Di formamide (0.89 µl) was loaded into the analyzer (Figure S1B–E).

**Sequencing library preparation**

The phi X174 phage genome and *E. coli* DNA (ATCC8739) were used to construct libraries following the procedure in Figure 1A. Since single-molecule sequencing (SMS) does not require amplification, adaptor ligation could be performed on only one end. To prevent the extension from the adaptor’s non-ligated 3' end, the 3' end of the adaptor DNA molecule was blocked with an amine linker. The libraries were diluted and then loaded into the flow cell with an automatic sampler. The flow cell was loaded into the SMS platform following the instructions to initiate sequencing.

**Surface hybridization**

The surface chemistry of the SMS platform’s flow cell has been described in previous publications [28,29]. Primers immobilized on the flow cell surface were designed to capture samples with sequencing adaptors. In the SMS platform system, a 16-lane flow cell was assembled with a functionalized glass coverslip (110 × 74 mm) and a bottom glass slide using pressure-sensitive adhesive. To maximize the uniformity and single-molecule ratio, we coated the coverslip with a single layer of epoxy silane by chemical vapor deposition. A 62-nt-long oligo with -NH_2_ modification at the 5' end was designed and anchored on the surface as a universal probe, while the primer could be customized according to specific applications. The probe contained ~ 50% GC, which could explicitly hybridize and stabilize sample DNA during sequencing. The library’s adaptor was modified with extra bases at the 3' position to prevent the extension from the 3' end of the library strand. Compared to the poly-T capture probe used in previous reports, this new design eliminated the fill-lock step and reduced the total sample loading time to 30 min. The design also allowed direct capture of the pre-selected genome region in targeted DNA sequencing.

**Sequencing reagent kit**

The GenoCare sequencing reagent kit contained two sets of nucleotide–polymerase mixtures, one for fluorescence dye-labeled A and T virtual terminators, and the other for G and C virtual terminators labeled with the same pair of dyes. It also included an image buffer, a cleave reagent, a blocking buffer, and two wash buffers. In each sequencing cycle, one set of nucleotide–polymerase mixture was added, and the dye-labeled terminator was incorporated by the polymerase. The image buffer was then introduced and the scan images were taken. The fluorescence dye was subsequently removed by the cleave reagent, and the thiol residue was deactivated by the blocking buffer. The wash buffers were applied to ensure complete replacement of each reagent from the flow cell after its corresponding reaction.

**Sequencer hardware**

The GenoCare platform (GeneMind, Shenzhen, China), a desktop platform for two-color SMS, was employed, which uses wide-field total internal reflection fluorescence (TIRF) optics to detect weak signals from single nucleotide molecules. In the TIRF system, green and red lasers with emission wavelengths of 532 nm and 639 nm, respectively, were utilized to simultaneously illuminate the samples through the edge of a high numerical aperture objective. The illumination generated an evanescent light wave layer as shallow as 100 nm beyond the glass–solution interface, which selectively excited the fluorescence labels of terminators on the surface while leaving most of the background unilluminated. To maximize the signal-to-noise ratio (SNR) of single molecules, the imaging system exploited two scientific complementary metal oxide semiconductor cameras with high quantum efficiency and low noise to photograph each field of view (FOV).

Image stabilization is a critical challenge for high-sensitivity single-molecule optics like TIRF. Single-molecule image quality is vulnerable to multiple sources, including environmental vibration, stage motion, temperature fluctuation, and air disturbance. Because the depth of field of the TIRF objective is as small as 500 nm, a high-accuracy auto-focusing system was used to lock the imaging focus in real-time during XY scanning of the flow cell. The mechanical structure of the instrument had a multiple-level anti-vibration design to minimize the effects of external and internal vibration sources. To speed up the sequencing process, the entire flow cell was divided into two parallel units with eight lanes each. During each sequencing cycle, when one unit was performing fluidic motion and biochemical reactions, the other performed imaging.

**Data processing**

The raw sequencing image files were analyzed in real-time by our in-house machine learning algorithm-based software (DirectCall 2C0.5.8). The data processing workflow included background elimination, spot localization, image registration, template building, and base-calling. To improve the mapping rate and shorten processing time, a Q-score system was employed to filter the raw data from base-calling. Unlike the Q-score system widely used by next-generation sequencing (NGS) platforms, the GenoCare SMS platform’s Q-score is based on the unique signal properties of the SMS and reflects the read quality defined by factors including signal intensity, SNR, and template distance. Higher Q-score DNA reads tend to show a lower error rate and higher mapping probability. A different Q-score can be used to filter raw data according to the application.

**Characterization of single-molecule sequencing**

Phi X174 phage genome DNA (single-stranded, circular genome) (Thermo Fisher Scientific, Waltham, Massachusetts) was sequenced as a standard reference template to characterize the reads yield of our platform. In consideration of the total sequencing time, we captured images from 16 lanes, with 500 FOVs in each lane, constituting 41.6% of the recommended maximum imaging area of the flow cell. We also hybridized E. coli DNA (ATCC8739, Guangdong Microbial Culture Collection Center) in one lane and sequenced 120 cycles with 400 FOVs captured from each cycle.

**Detection of microbes**

To evaluate the sequencing accuracy for each sample, we constructed 14 libraries by mixing pure *E. coli* DNA (ATCC8739) and healthy female individual genomic DNA to obtain different ratios of *E. coli*(Table S5). Furthermore, we mixed E. coli DNA (ATCC8739), S. aureus DNA [CMCC(B)26003], M13mp18 RF I DNA (a circular form of phage M13 DNA), yeast DNA, and human DNA from the healthy female individual at different ratios (Table S2). The DNA mixture was split into two aliquots, one for the SMS platform and the other for the NGS sequencer (HiSeq 4000, Illumina, San Diego, California) in SE150 mode. For SMS, we sequenced 72 cycles with 480 FOVs captured from each cycle. The data for the first 40 bp from the NGS sequencer were selected for parallel comparison with the GenoCare SMS platform. As to the 6 M unique reads, the ratios for the detected unique reads from E. coli, S. aureus, M13, and yeast on both the SMS and NGS sequencers were calculated. Specific primers were designed to quantify S. aureus and M13 at different ratios by real-time PCR (CFX96 Touch, Bio-Rad, Hercules, California).
