# Supplementary material for: A Two-color Single-molecule Sequencing Platform and Its Clinical Applications
Source: Genomics Proteomics Bioinformatics. 2024 Jan 11;22(1):qzae006. doi: 10.1093/gpbjnl/qzae006 (PMC11423845; doi:10.1093/gpbjnl/qzae006)
Supplement: qzae006_Supplementary_Data [file qzae006_supplementary_data.zip › Supplementary captions.docx]

**Supplementary material**

**File S1 Extended experimental procedure**

**Figure S1** **Terminator reaction in bulk solution**

**A.** Terminator structure. **B.** Capillary electrophoresis analysis results for the reaction without terminator. **C.** Capillary electrophoresis analysis results for reaction of terminator A with the first triple T template. **D.** Capillary electrophoresis analysis results for reaction of terminator A with the first T and second C template. **E.** Terminator A reacted with the first T and second A template.

**Figure S2 Error rates between single-color and updated two-color sequencing chemistry**

**A.** Substitution errors. **B.** Three other error types.

**Figure S3 Unique mapped reads of phi X174 samples in 16 lanes**

The phi X174 sample was hybridized and sequenced in a 16-lane flow cell using 72 cycles.

**Figure S4 Test reads ratio of three microorganisms on the GenoCare and HiSeq4000 sequencers**

**A.** *Saccharomyces cerevisiae*. **B.** *Staphylococcus aureus*. **C.** *E*. *coli*.

**Figure S5 Mapping reads of positive and negative samples across the SARS-CoV-2 genome**

**A**, **B**, **C**, and **D** indicate data from patient samples 1016T, 1022T, and 1028T, and the negative control sample. The total unique read numbers for positive samples are 0.036 million (1016T), 0.53 million (1022T), and 0.24 million (1028T), with average sequencing depths of 40×, 660×, and 300×, respectively. SARS-CoV-2, severe acute respiratory syndrome coronavirus 2.

**Figure S6 Library preparation process for SARS-CoV-2 sample using the transposome method**

The libraries of RNA samples from throat swabs were prepared using the Tn5 transposome method. Following reverse transcription, the Tn5 transposome could randomly bind RNA/DNA heteroduplex and add sequencing adaptors. The complete libraries were obtained by PCR. PCR, polymerase chain reaction.

**Table S1 Sequencing characteristics of single-color and updated two-color sequencing chemistry**

**Table S2 Theoretical mass ratio of the microbial genome in sample mixtures**

**Table S3 Detected mutations in SARS-CoV-2 (1022T sample) confirmed by Sanger sequencing**

**Table S4 Detected mutations in SARS-CoV-2 (1028T sample) confirmed by Sanger sequencing**

**Table S5 The results of the pure concentration gradient experiment**
