# Supplementary material for: A Two-color Single-molecule Sequencing Platform and Its Clinical Applications
Source: Genomics Proteomics Bioinformatics. 2024 Jan 11;22(1):qzae006. doi: 10.1093/gpbjnl/qzae006 (PMC11423845; doi:10.1093/gpbjnl/qzae006)
Supplement: qzae006_Supplementary_Data [file qzae006_supplementary_data.zip › Table S1.docx]

**Table S1 Sequencing characters of single-color and updated two-color sequencing chemistry**

|  | **Cycle number** | **Average read length (nt)** | **Substitution ratio (%)** | **Insertion ratio (%)** | **Deletion ratio (%)** | **Total error ratio (%)** |
| --- | --- | --- | --- | --- | --- | --- |
| Single color | 120 | 38 | 0.84 | 2.81 | 2.68 | 6.33 |
| Two-color | 72 | 41 | 0.83 | 1.92 | 3.10 | 5.85 |
