# Supplementary material for: A Two-color Single-molecule Sequencing Platform and Its Clinical Applications
Source: Genomics Proteomics Bioinformatics. 2024 Jan 11;22(1):qzae006. doi: 10.1093/gpbjnl/qzae006 (PMC11423845; doi:10.1093/gpbjnl/qzae006)
Supplement: qzae006_Supplementary_Data [file qzae006_supplementary_data.zip › Table S2.docx]

**Table S2 Theoretical mass ratio of the microbial genome in sample mixtures**

| **Sample** | **Total mass** | **Human genome** | | **Yeast genome** | | ***E. coli* genome (ATCC8739)** | | ***S. aureus* genome [CMCC(B) 26003]** | | **M13mp18 RF I DNA** | |
| --- | --- | --- | --- | --- | --- | --- | --- | --- | --- | --- | --- |
|  |  | **Mass ratio** | **Input mass (ng)** | **Mass ratio** | **Input mass (ng)** | **Mass ratio** | **Input mass (ng)** | **Mass ratio** | **Input mass (ng)** | **Mass ratio** | **Input mass (ng)** |
| PM_A | 10000 | 99.79989% | 9979.99 | 1×10^-3^ | 10.00 | 1×10^-6^ | 0.01 | 1×10^-3^ | 10.00 | 1×10^-7^ | 0.001 |
| PM_B | 10000 | 99.8994% | 9989.94 | 5×10^-4^ | 5.00 | 5×10^-6^ | 0.05 | 5×10^-4^ | 5.00 | 1×10^-6^ | 0.010 |
| PM_C | 10000 | 99.9785% | 9997.85 | 1×10^-4^ | 1.00 | 1×10^-5^ | 0.10 | 1×10^-4^ | 1.00 | 5×10^-6^ | 0.050 |
| PM_D | 10000 | 99.984% | 9998.40 | 5×10^-5^ | 0.50 | 5×10^-5^ | 0.50 | 5×10^-5^ | 0.50 | 1×10^-5^ | 0.100 |
| PM_E | 10000 | 99.983% | 9998.30 | 1×10^-5^ | 0.10 | 1×10^-4^ | 1.00 | 1×10^-5^ | 0.10 | 5×10^-5^ | 0.500 |
| PM_F | 10000 | 99.939% | 9993.90 | 5×10^-6^ | 0.05 | 5×10^-4^ | 5.00 | 5×10^-6^ | 0.05 | 1×10^-4^ | 1.000 |
| PM_G | 10000 | 99.7998% | 9979.98 | 1×10^-6^ | 0.01 | 1×10^-3^ | 10.00 | 1×10^-6^ | 0.01 | 1×10^-3^ | 10.000 |

*Note*: *E*. *coli*, *Escherichia coli*; *S*. *aureus*, *Staphylococcus aureus*.
