# Supplementary material for: A Two-color Single-molecule Sequencing Platform and Its Clinical Applications
Source: Genomics Proteomics Bioinformatics. 2024 Jan 11;22(1):qzae006. doi: 10.1093/gpbjnl/qzae006 (PMC11423845; doi:10.1093/gpbjnl/qzae006)
Supplement: qzae006_Supplementary_Data [file qzae006_supplementary_data.zip › Table S3.docx]

**Table S3 Detected mutations of SARS-CoV-2 (1022T sample) confirmed by Sanger sequencing**

| **Primer** |  | **Ref.** | **Mutant** |
| --- | --- | --- | --- |
| P1 | F:5'-GACCTGAAGGGATACCACGAT-3'  R:5'-CTCAAGCTGGTTCAATCTGTCA-3' | ATTCA**a**GGCTCCCT  Position: 28,688 | ATTCA**g**GGCTCCCT  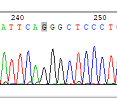 |
| P2 | F:5'-AGACAGATAGGTCAACGCAGT-3'  R:5'-ATTCATTATGGTATTCGGCAAG-3' | TCAGAA**g**TAGGACCT  Position: 1397 | TCAGAA**a**TAGGACCT  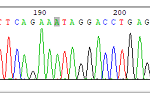 |
| P3 | F 5'-TAAGAGCATTGATGTATCGTGT-3'  R 5'-TAAAATCACATGGGGATAGCAC-3' | GGCCAC**g**CGGAGTAC  Position: 29,742 | GGCCAC**t**CGGAGTAC  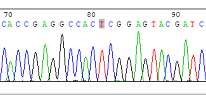 |
| P4 | F:5'-TTCTTGTTAGTTCCCATGTGT-3'  R:5'-CTAGCAGGCATATAGACCA-3' | ATTTTCATA**c**AAAAAAAA  Position: 11,083 | ATTTTCATA**a**AAAAAAAA  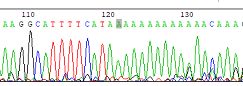 |
| P5 | F:5'-TAAGAGCATTGATGTATCGTGT-3'  R:5'-TAAAATCACATGGGGATAGCAC-3' | ACAATGCT**a**GGGAGAGC  Position: 29,776 | ACAATGCT**t**GGGAGAGC  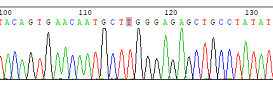 |

*Note*: SARS-CoV-2, severe acute respiratory syndrome coronavirus 2.
