# Supplementary material for: A Two-color Single-molecule Sequencing Platform and Its Clinical Applications
Source: Genomics Proteomics Bioinformatics. 2024 Jan 11;22(1):qzae006. doi: 10.1093/gpbjnl/qzae006 (PMC11423845; doi:10.1093/gpbjnl/qzae006)
Supplement: qzae006_Supplementary_Data [file qzae006_supplementary_data.zip › Table S4.docx]

**Table S4 Detected mutations of SARS-CoV-2 (1028T sample) confirmed by Sanger sequencing**

| **Primer** |  | **Ref.** | **Mutant** |
| --- | --- | --- | --- |
| P1 | F:5'-ACTTTAGTATCCTATGTTCCG-3'  R:5'-TCACCATTAGTTGTGCGTA-3' | ACGCTG**g**CTAAACC  Position: 8782 | ACGCTG**a**CTAAACC  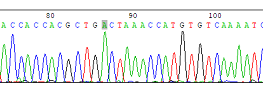 |
| P2 | F:5'-GACCTGAAGGGATACCACGAT-3'  R:5'-CTCAAGCTGGTTCAATCTGTCA-3' | AGGCAGCA**g**TAGGGGA  Position: 28,878 | AGGCAGCA**a**TAGGGGA  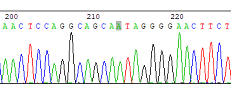 |
| P3 | F:5'-ACTGGGCACAGGATAAGTGA-3'  R:5'-CGCACTACAAGACTACCCAA-3' | TTTCCTGTT**t**ACCTTTTA  Position: 28,144 | TTTCCTGTT**c**ACCTTTTA  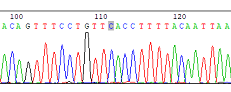 |
| P4 | F:5'-TAATGCGTGTTGATTACCACT-3'  R:5'-GGCGTAAACTTTCATAAGCA-3' | TTACCAACT**g**CACTAAA  Position: 8937 | TTACCAACT**a**CACTAAA  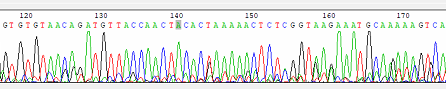 |
| P5 | F:5'-TAAGAGCATTGATGTATCGTGT-3'  R:5'-TAAAATCACATGGGGATAGCAC-3' | AGGCCAC**g**CGGAGTA  Position: 29,742 | AGGCCAC**a**CGGAGTA  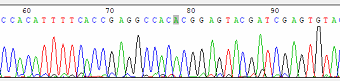 |

*Note*: SARS-CoV-2, severe acute respiratory syndrome coronavirus 2.
