# Supplementary material for: A Two-color Single-molecule Sequencing Platform and Its Clinical Applications
Source: Genomics Proteomics Bioinformatics. 2024 Jan 11;22(1):qzae006. doi: 10.1093/gpbjnl/qzae006 (PMC11423845; doi:10.1093/gpbjnl/qzae006)
Supplement: qzae006_Supplementary_Data [file qzae006_supplementary_data.zip › Table S5.docx]

**Table S5 The result of the pure concentration gradient experiment**

| **Lane name** | **Sample** | ***E. coli*** DNA mass ratio (%) | **Total unique reads number** | **Mapping** E. coli reads number | ***E. coli*** reads ratio (%) |
| --- | --- | --- | --- | --- | --- |
| L01 | ECOLI_A_1 | 0.0001 | 8,425,118 | 35 | 0.0004 |
| L02 | ECOLI_A_2 | 0.0001 | 8,417,293 | 25 | 0.0003 |
| L03 | ECOLI_B_1 | 0.0005 | 8,363,756 | 40 | 0.0005 |
| L04 | ECOLI_B_2 | 0.0005 | 8,647,415 | 50 | 0.0006 |
| L05 | ECOLI_C_1 | 0.001 | 8,390,434 | 82 | 0.001 |
| L06 | ECOLI_C_2 | 0.001 | 8,525,356 | 58 | 0.001 |
| L07 | ECOLI_D_1 | 0.005 | 8,332,254 | 269 | 0.003 |
| L08 | ECOLI_D_2 | 0.005 | 8,280,753 | 309 | 0.004 |
| L09 | ECOLI_E_1 | 0.01 | 8,569,052 | 577 | 0.01 |
| L10 | ECOLI_E_2 | 0.01 | 8,478,103 | 634 | 0.01 |
| L11 | ECOLI_F_1 | 0.05 | 8,682,213 | 3,207 | 0.04 |
| L12 | ECOLI_F_2 | 0.05 | 8,771,663 | 3,366 | 0.04 |
| L13 | ECOLI_G_1 | 0.1 | 9,211,204 | 7,121 | 0.1 |
| L14 | ECOLI_G_2 | 0.1 | 7,531,005 | 5,711 | 0.1 |

*Note*: *E. coli* DNA mass ratio = *E. coli* / (*E. coli* + Human). Mapping *E.coli* reads number means the reads number mapped to the genome of *E.coli* ATCC8739. *E.coli* reads ratio = Mapped *E.coli* reads number / Total unique reads.
