# Supplementary figures and images for: A Two-color Single-molecule Sequencing Platform and Its Clinical Applications
Source: Genomics Proteomics Bioinformatics. 2024 Jan 11;22(1):qzae006. doi: 10.1093/gpbjnl/qzae006 (PMC11423845; doi:10.1093/gpbjnl/qzae006)

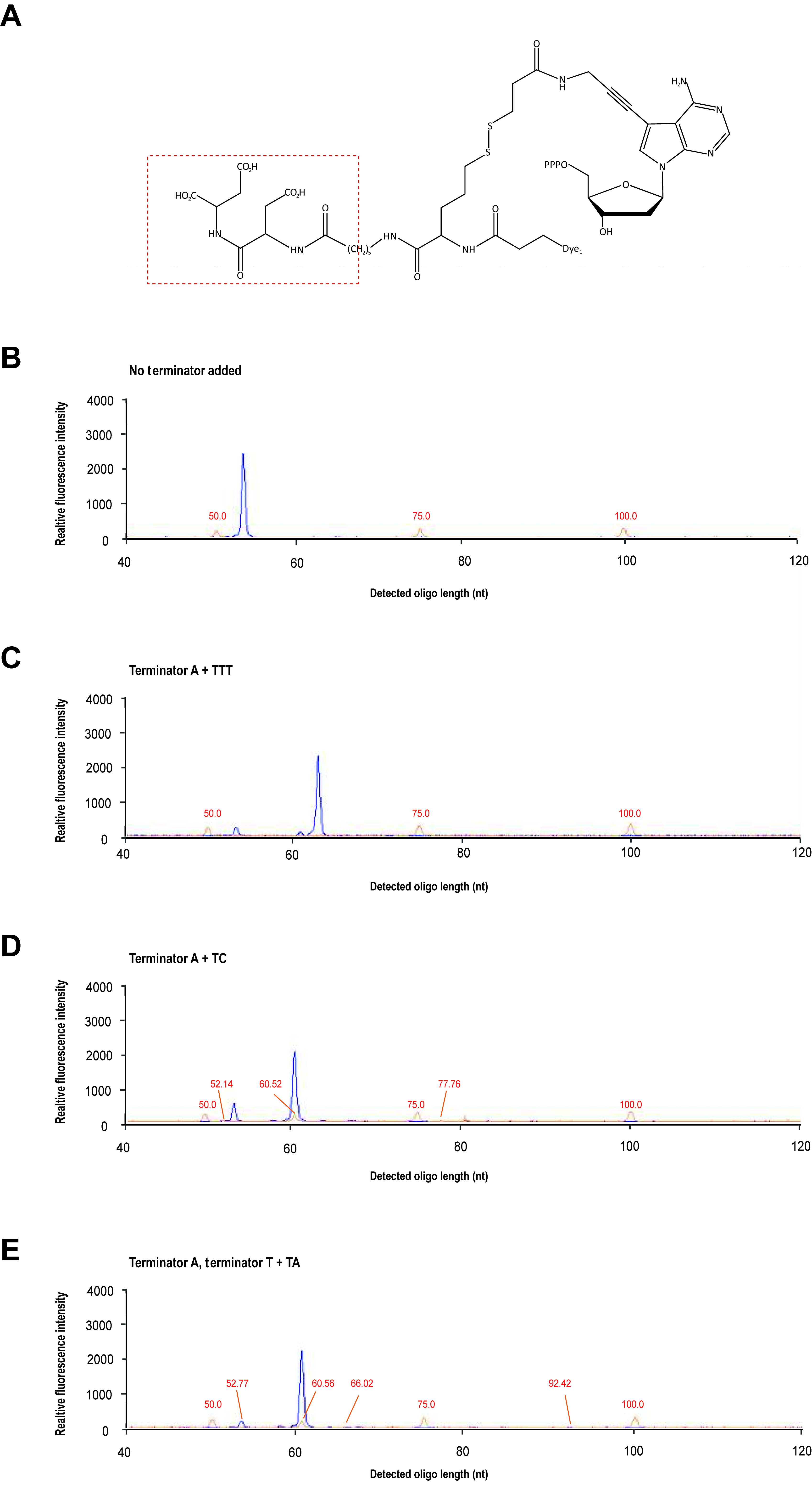

Supplement: qzae006_Supplementary_Data [file qzae006_supplementary_data.zip › Figure S1.tif]

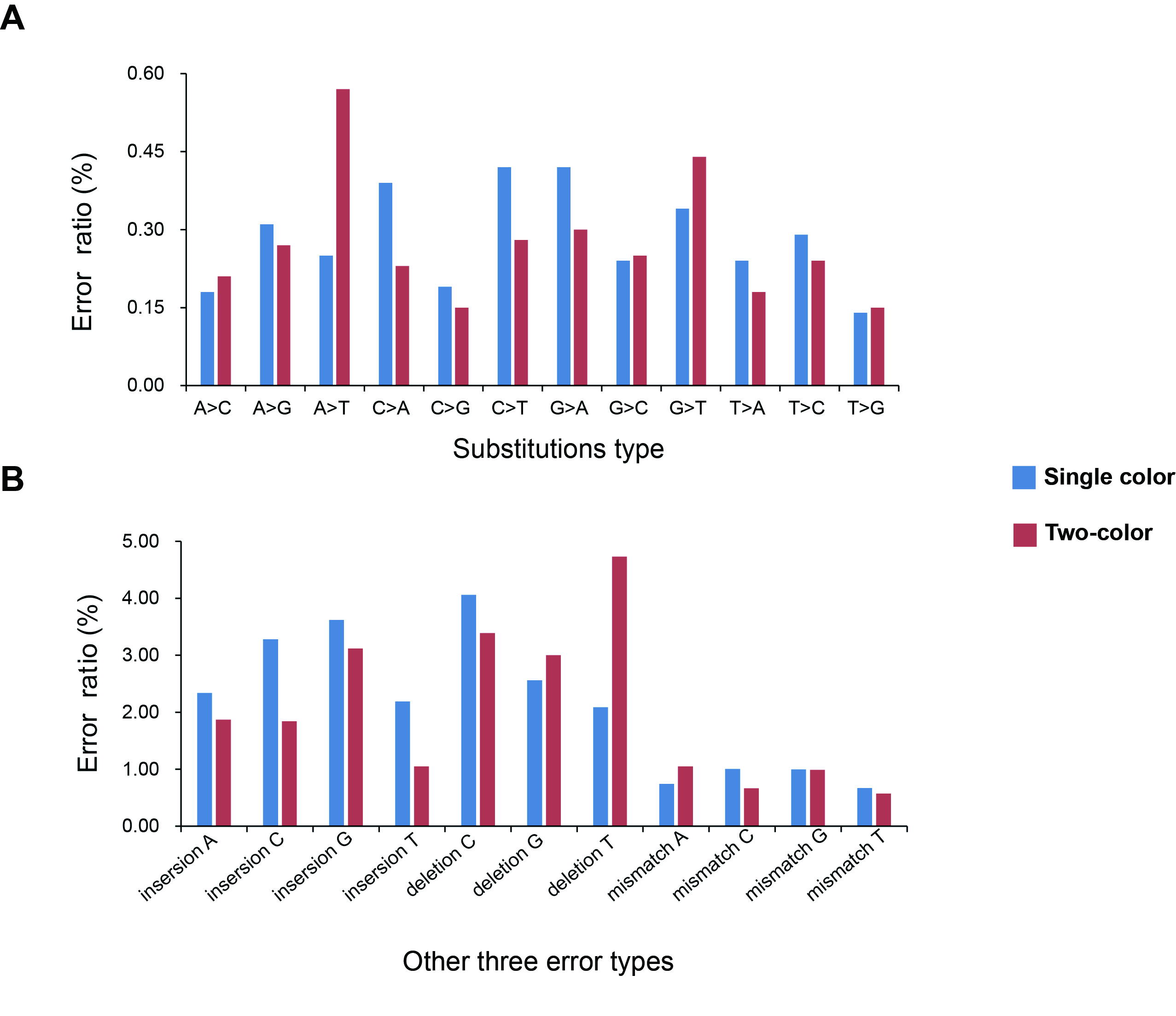

Supplement: qzae006_Supplementary_Data [file qzae006_supplementary_data.zip › Figure S2.tif]

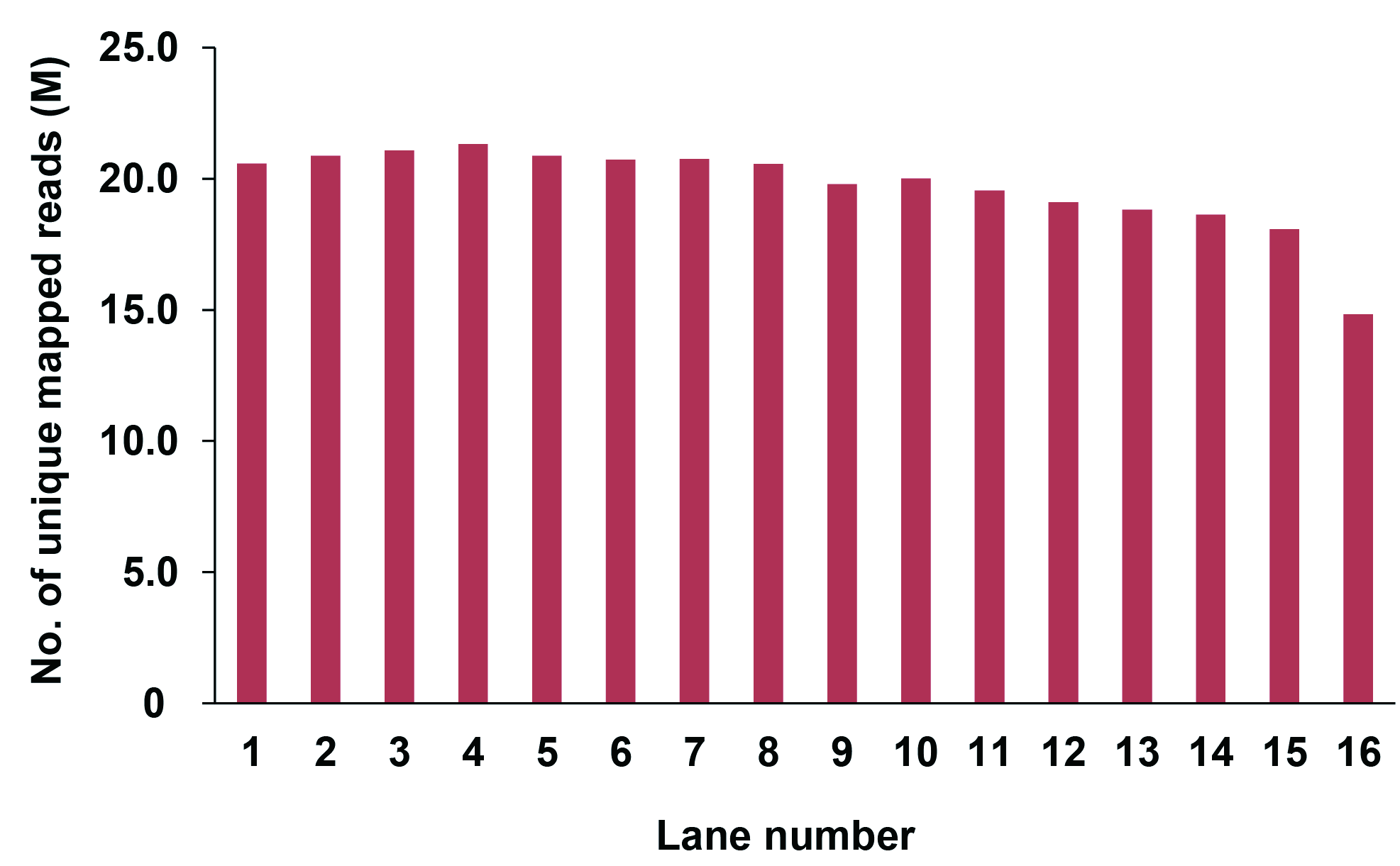

Supplement: qzae006_Supplementary_Data [file qzae006_supplementary_data.zip › Figure S3.tif]

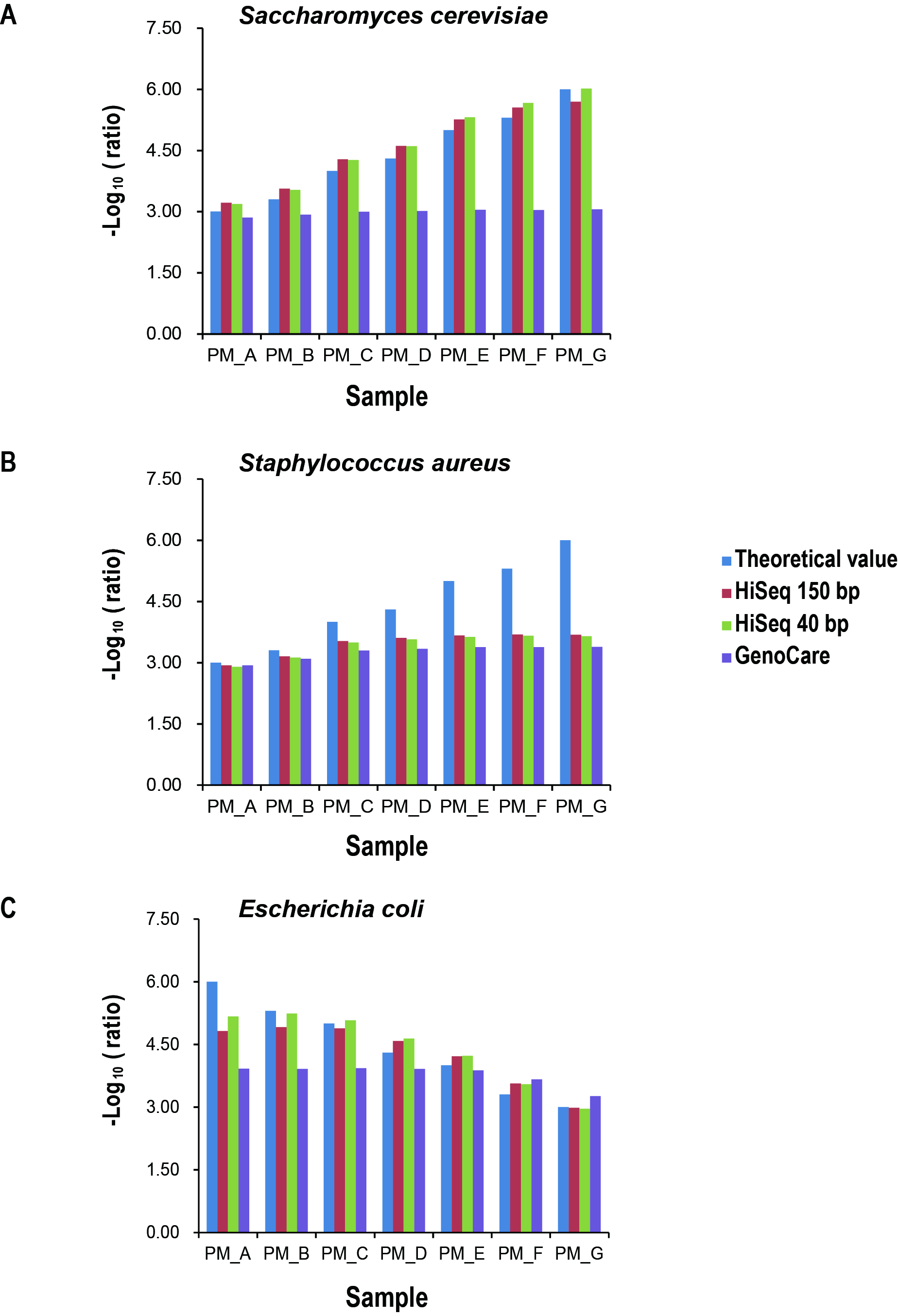

Supplement: qzae006_Supplementary_Data [file qzae006_supplementary_data.zip › Figure S4.tif]

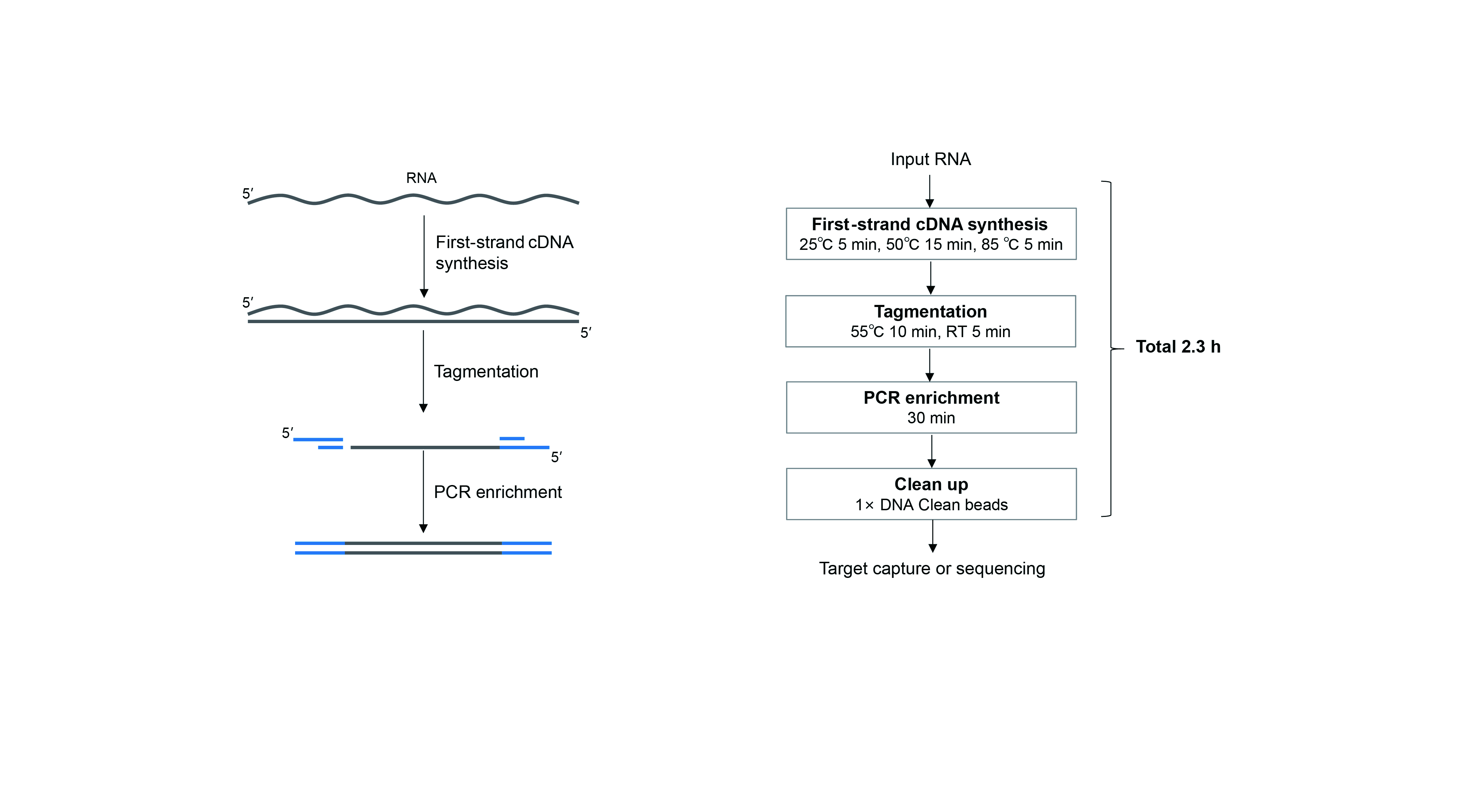

Supplement: qzae006_Supplementary_Data [file qzae006_supplementary_data.zip › Figure S6.tif]
